# Structure of Supramolecular Assemblies Formed by α,δ-Tetramethylcucurbit[6]uril and 4-Nitrophenol

**DOI:** 10.3390/molecules13112814

**Published:** 2008-11-12

**Authors:** Li-Mei Zheng, Yun-Qian Zhang, Jin-Ping Zeng, Yan Qiu, Da-Hai Yu, Sai-Feng Xue, Qian-Jiang Zhu, Zhu Tao

**Affiliations:** 1 School of Chemistry and Chemical Engineering, Henan University of Technology, Zhengzhou 450001, People’s Republic of China; 2 Key Laboratory of Macrocyclic and Supramolecular Chemistry of Guizhou Province, Guizhou University, Guiyang 550025, People’s Republic of China; 3 Institute of Applied Chemistry, Guizhou University, Guiyang 550025, People’s Republic of China

**Keywords:** α,δ-Tetramethylcucurbit[6]uril (TMeQ[6]), 4-Nitrophenol, Exclusion host-guest assembly, Driving forces.

## Abstract

A host-guest assembly, [(C_40_H_44_N_24_O_12_)·(C_6_H_5_NO_3_)_8_·13(H_2_O)] (**1**), based on a partial substituted cucurbituril, α,δ-tetramethylcucurbit[6]uril (TMeQ[6]), and 4-nitrophenol was synthesized and structurally characterized by single-crystal X-ray diffraction. A combination of hydrogen-bonding between the latticed water molecule and the hydroxyl group of 4-nitrophenol, the hydroxyl group of 4-nitrophenol and the carbonyl groups lining the portals in additon, the C-H···π interactions between the 4-nitrophenol molecules could be the driving forces of formation such an exclusion host-guest assembly.

## Introduction

The chemistry of cucurbit[n]urils (Q[n]s) has been studied extensively [1] since the first member of the family, cucurbituril, was structurally characterized by Mock and coworkers in 1981 [2]. The cucurbit[n]urils include cucurbituril (Q[6]) and its homologues, cucurbit [n = 5, 7, 8 and 10]urils (Q[5], Q[7], Q[8] and Q[10]) [3,4,5], and a series of fully or partially substituted derivatives and analogues [6,7,8,9,10,11,12]. The members of the cucurbit[n]uril family have common characteristic features, such as a hydrophobic cavity and polar carbonyl groups surrounding the opening portals. In addition, their varying cavity and portal sizes lead to the formation of inclusion or exclusion complexes with different organic or inorganic species. They also present remarkable molecular recognition properties and act as building blocks for supramolecular chemistry [5,13,14,15,16,17,18]. Up to now, the supramolecular assemblies of metal or cluster aqua complexes involving the Q[n]s have been mainly limited in those with Q[6] or Q[8]. After Mock and Kim’s research [19,20], Fedin and coworkers have done major work in this area and their results were summarized in reviews [21,22]. They found that alkali and alkaline earth metal ions are coordinated directly with carbonyl oxygen atoms of Q[6], but transition metal ions prefer to form hydrogen bonds between the polar C=O groups of Q[6].

**Figure 1:**
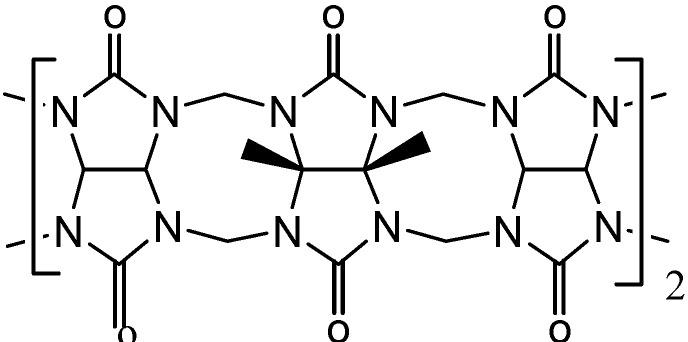
Structure of TMeQ[6].

We previously reported the controlled synthesis of α, δ-tetramethylcucurbit[6]uril (TMeQ[6]) and the preparation of some supramolecular assemblies of metal aqua complexes and host-guest inclusion complexes involving this substituted Q[n]s (Figure 1)[11]. In this work, we report the crystal structures of an exclusion supramolecular assembly of TMeQ[6] with 4-nitrophenol

## Results and Discussion

The X-ray structure of TMeQ[6] shows that it is a macrocyclic cavity with two identical portals surrounded by carbonyl groups. Unlike the normal cucurbit[n=5-10]urils, the α,δ-substituted Q[6] macrocycle is not circular, but ellipsoid. Its structure is easy to deform and includes molecules which could not be included by the normal cucurbit[6]uril [23]. 

**Figure 2:**
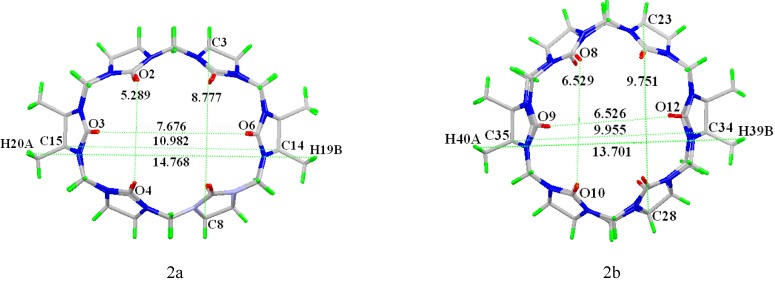
Two different styles of TMeQ[6] molecules (a) Q1 and (b) Q2.

In the crystal structure of an exclusion host-guest assembly of TMeQ[6] with 4-nitrophenol, the phenyl moiety of 4-nitrophenol was not included in the TMeQ[6] cavity due to some unfavorable interactions between the carbonyl oxygens of the TMeQ[6] and the 4-nitrophenol nitro group. However, an excluding interaction between the carbonyl oxygens of the TMeQ[6] and hydroxyl group of 4-nitrophenol was observed in compound **1**. There were two different styles of TMeQ[6] molecules, one included four water molecules (marked Q1), while the other included nothing in the cavity (marked Q2). The structural characteristics of the two styles of TMeQ[6] were obviously different. As shown in Figure 2a the Q1 is ellipsoid, and the distance between the O3 and O6 portal oxygens is about 7.676 Å, while the distance between the portal oxygens O2 and O4 is only 5.289 Å. The distance between the cavity carbons C14 and C15 is about 10.982 Å, while the distance between the cavity carbons C3 and C8 is about 8.777 Å. The distance between H20A and H19B is about 14.768 Å. For the Q2 (Figure 2 b), the corresponding distance between the portal oxygens O9 and O12 is about 6.526 Å, the corresponding distance between the portal oxygens O8 and O10 is 6.529 Å. The distance between the cavity carbons C34 and C35 is about 9.955 Å, while the distance between the cavity carbons C23 and C28 is about 9.751 Å. The distance between H40A and H39B is about 13.701 Å.

As mentioned above, TMeQ [6] is easy to be deformed, and the different deformation of the two types of TMeQ [6] in the title compound could be caused by the different interactions with included water molecules, excluded 4-nitrophenol molecules or latticed water molecules. For the Q1, four water molecules O12W, O12W^i^, O13W and O13W^i^ form a diamond plane in the cavity of the TMeQ[6] (symmetry codes: (i) 1-x, 1-y, -z). Both portals of Q1 are covered a “net” formed through the hydrogen-bonding of carbonyl oxygens and the latticed water molecules, in addition, the hydrogen-bonding between the latticed water molecules, such as, O3W···O3 (2.662Å), O3W···O2 (2.841Å), O5W···O5 (2.805Å), O1W···O6 (2.665Å), and O1W···O5W (2.716 Å), O5W···O3W (2.715 Å). Thus, a molecular encapsulate of TMeQ[6] with four included water molecules and the two covered nets is formed. Both portals of Q2 are also covered a “net” formed through the hydrogen-bonding of carbonyl oxygens and the latticed water molecules, in addition, the hydrogen-bonding between the latticed water molecules, such as, O9W···O7 (2.904 Å), O9W···O12 (2.927 Å), O14W···O9 (2.694 Å), O14W···O9W (2.094 Å)O10W···O10(2.800Å), O10W···O11(2.817Å) and O10W···O9W(2.778Å). Moreover, a 4-nitrophenol interacts with Q2 through the hydrogen-bonding of O16···O8 (2.733 Å). In this case, one can only see a molecular encapsulate shell.

**Figure 3:**
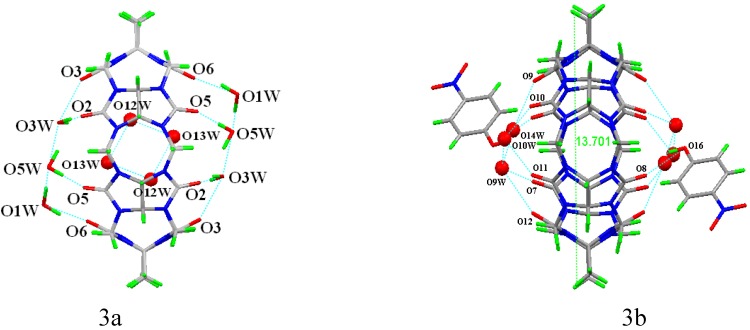
The molecular encapsulate based on the Q1 (a), and the the molecular encapsulate shell based on the Q2 (b).

**Figure 4:**
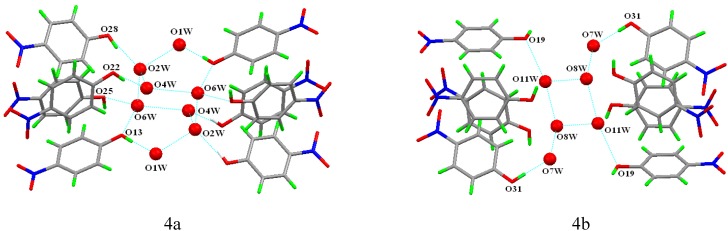
Two different 4-nitrophenol assemblies(4a) **A1** and (4b) **A2**.

In the crystal structure of compound **1**, the guest 4-nitrophenol was not included in the cavity of the host TMeQ[6]. However, every eight 4-nitrophenol molecules assembled through not only the hydrogen bonding of the latticed water molecules, but also the C-H···π interaction, and there are two different 4-nitrophenol assemblies. In the first 4-nitrophenol assembly (**A1**), a chair shape cyclic cluster consists of six water molecules O2W, O2W^ii^, O4W, O4W^ii^, O6W O6W^ii^ (symmetry codes: (ii) 1-x, 1-y, 1-z) through the hydrogen-bonding, such as O2W···O6W (2.760 Å), O4W···O2W (2.828 Å), O6W···O4W (2.960 Å), every water molecule of this six member ring connects a 4-nitrophenol molecule, except the water molecule O6W, which connects two 4-nitrophenol molecules through the hydrogen-bonding, such as O22···O4W(2.650Å), O28···O2W (2.708Å), O6W···O25 (3.011Å), O6W···O13 (2.865Å), in addition, the lattice water molecule O1W connects a 4-nitrophenol molecule and other water molecule O2W. Thus, the eight 4-nitrophenol molecules are assembled together in a tail to tail manner (Figure 4a). However, in the second 4-nitrophenol assembly (**A2**), six lattice water molecules O7W, O7W^iii^, O8W, O8W^iii^, O11W O11W^iii^ (symmetry codes: (iii) 1-x, 1-y, 1-z) connect four 4-nitrophenol molecules of the second assembly through hydrogen bonding, such as O11W···O19(2.894Å), O31···O7W (2.624Å), and the rest four 4-nitrophenol molecules in the second assembly seem to be independent (Figure 4b).

**Figure 5:**
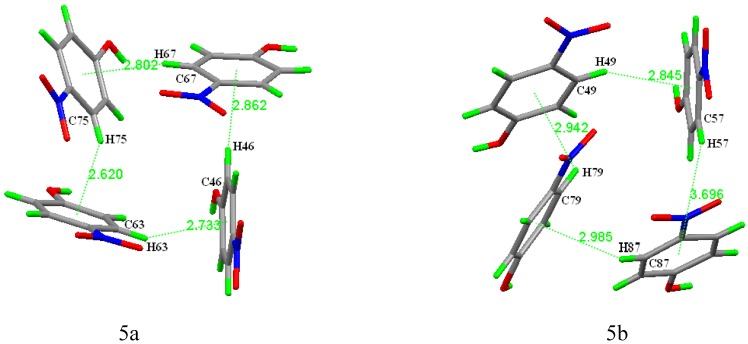
The C-H···π structures in the two different 4-nitrophenol assemblies.

In both 4-nitrophenol assemblies, we did not observed the π-π stacking which normally occurred in the crystal structure of aromatic compounds, but the C-H···π structure [24,25,26]. In the structure, there are eight C-H···π structures and each of them is symmetric (Table 1, Figure 5).

**Table 1:** The C-H…\p interactions (**Å, º**).

C-H…π	C-H(Å)	H…Cg(Å)	C…Cg(Å)	C-H…Cg(Å)	aromatic ring	symmetry codes
C46-H46…Cg(1)	0.93	2.862	3.647(3)	120.27	C65-C70	
C67-H67…Cg(2)	0.93	2.802	3.629(3)	148.80	C71-76	x, y, 1+z
C75-H75…Cg(3)	0.93	2.620	3.316(4)	132.14	C59-C64	
C63-H63…Cg(4)	0.93	2.733	3.533(3)	144.68	C41-C46	x, y, -1-z
C57-H87…Cg(5)	0.93	3.693	4.332(4)	128.03	C83-C88	
C87-H87…Cg(6)	0.93	2.985	3.601(3)	125.10	C77-C82	
C79-H79…Cg(7)	0.93	2.942	3.499(3)	119.81	C47-C52	
C49-H49…Cg(8)	0.93	2.645	3.696(3)	152.80	C53-C58	1-x, 1-y, 1-z

It is conceivable that the C-H···π interaction could be a driving force of formation of the 4-nitrophenol assembly although no hydrogen-bonding exist between the latticed water molecules and the 4-nitrophenol molecules in the **A2**. The C-H···π interaction occurring in the **A1** could further strengthen the 4-nitrophenol assembly in this case.

**Figure 6:**
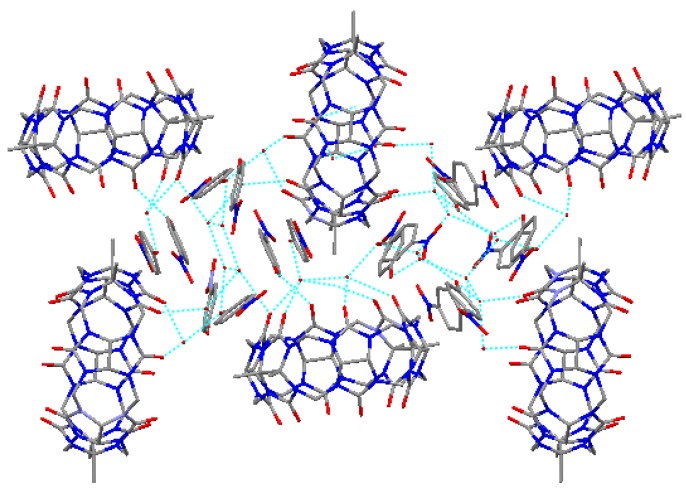
The assembly based on TMeQ[6] and 4-nitrophenol in the compound **1**.

Here we presented an assembly based on TMeQ[6] and 4-nitrophenol, where several main interactions could be the diving forces of the formation of this exclusion host-guest assembly. First, the hydrogen-bonding of carbonyl oxygens of TMeQ[6] and the latticed water molecules, in addition, the hydrogen-bonding between the latticed water molecules. Second, the hydrogen bonding of the hydroxyl group of 4-nitrophenol and the latticed water molecules. Third, the C-H···π interactions between 4-nitrophenol molecules. Thus, in the assembly of the title compound, the two different TMeQ[6] (Q1 and Q2) array alternatively, and the assemblies (**A1** and **A2**) scatter in the spaces of the lattice of TMeQ[6] (refer to Figure 6).

## Experimental

### General

4-Nitrophenol was of reagent grade and used without further purification. The TMeQ[6] was prepared by procedures reported previously [11]. Elemental analysis was carried out on 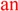 EURO EA-3000 element analyzer.

### Preparation of (TMeQ[6]-4nitrophenol)·13H_2_O (1)

Single crystals of the Me4Q[6] adduct with 4-nitrophenol were obtained by dissolving Me4Q[6] (0.50 g, 0.47 mmol) in a solution of 4-nitrophenol (0.53 g, 3.81 mmol) in water (10 mL). The final solution was mixed thoroughly and allowed to stand at room temperature; crystals formed after several days, and were collected.

### X-ray Crystallography

The crystal data of the substituted cucurbiturils coordinated with metal ions were collected on a SMART ApexII CCD diffractometer with graphite monochromated Mo Kα radiation ( λ = 0.71073 Å) in the *ω-φ* scan mode. Lorentz polarization and absorption corrections were applied. Structural solution and full matrix least-squares refinement based on *F*^2^ were performed with the SHELXS-97 and SHELXL-97 program package[27], respectively. All the non-hydrogen atoms were refined anisotropically. Analytical expressions of neutral-atom scattering factors were employed, and anomalous dispersion corrections were incorporated. The crystallographic data, data collection conditions, and refinement parameters for the compound **1** are listed in Table 2. 

**Table 2:** Crystallographic data for complex **1**.

Empirical formula	C_88_H_110_N_32_O_49_	Dcalcd, g cm^-3^	1.491
Formula weight	2400.08	T, K	223
Crystal system	Monoclinic	μ, mm-1	0.123
Space group	P-1	unique reflns	18398
a, Å	17.758(6)	obsdreflns	11799
b, Å	17.908(6)	params	1533
c, Å	18.941(7)	Rint	0.0430
α,deg	88.290(5)	R[I> 2σ(I)]*	0.0828
β,deg	83.072(5)	wR[I> 2σ(I)]#	0.2562
γ,deg	63.602(5)	R(all data)	0.1228
V, Å^3^	5346	WR(all data)	0.2786
Z	2	GOF on F2	1.052

^* ^Conventional *R* on F*hkl*: Σ||*F*o| - |*F*c||/Σ|*F*o|. ^# ^Weighted *R* on |F*hkl*|^2^: {Σ[*w*(*F*o^2^-*F*c^2^)^2^]/Σ[*w*(*F*o^2^)^2^]}^1/2^

Crystallographic data (excluding structure factors) for the structure reported in this paper have been deposited with the Cambridge Crystallographic Data Centre as Deposition No. CCDC 659590. Copies of the data can be obtained free of charge on application to CCDC, 12 Union Road, Cambridge CB2 1EZ, UK (Fax: + 44-1223/336-033; E-mail: deposit@ccdc.cam.ac.uk).

## Supporting Information Available

Experimental details of X-ray crystal structure determination, tables of X-ray data collection/refinement parameters, atomic position parameters, and anisotropic displacement parameters for **1**.
